# Embryonal tumors with multilayered rosettes, *C19MC*-altered or not elsewhere classified: Clinicopathological characteristics, prognostic factors, and outcomes of 17 children from 2018 to 2022

**DOI:** 10.3389/fonc.2022.1001959

**Published:** 2022-10-24

**Authors:** Kailun Xu, Zhaoyun Sun, Lifeng Wang, Wenbin Guan

**Affiliations:** ^1^ Department of Pathology, Xin Hua Hospital Affiliated to Shanghai Jiao Tong University School of Medicine, Shanghai, China; ^2^ Department of Cardiothoracic Surgery, Second Clinical Medical College, Nanjing Medical University, Nanjing, China

**Keywords:** embryonal tumors with multilayered rosettes, immunohistochemistry, molecular genetics, *C19MC*, Lin28A

## Abstract

**Objective:**

Embryonal tumors with multilayered rosettes (ETMRs) are a histologically heterogeneous entity and gather embryonal tumors with abundant neuropil and true rosettes (ETANTRs), ependymoblastoma, and medulloepithelioma. ETMRs are highly aggressive and associated with poorer clinical courses. However, cases of this entity are rare, and advances in molecular genetics and therapy are minor. The purpose of our study was to retrospectively analyze the clinical, pathological features, and prognostic factors of ETMRs.

**Methods:**

Our cohort consisted of 17 patients diagnosed with ETMRs in our hospital from 2018 to 2022, and two of them were lost to follow-up. Clinical data were retrieved, and immunohistochemistry and genetic analyses were performed.

**Results:**

Among 17 cases, 16 were ETANTRs, and one was medulloepithelioma. Morphologically, tumor cells of ETANTRs could transform into anaplasia and lose the biphasic architecture during tumor progression. Immunohistochemistry of LIN28A revealed positive expression in 17 cases, and the expression of LIN28A was more intense and diffuse in the recurrent lesions than in primaries. The increased *N-MYC* copy numbers were detected in the primary tumor and recurrence of patient 8. Moreover, the incidence of metastatic disease was 100% in patients aged > 4 years and 18% in the younger group. For patients receiving chemotherapy, the median overall survival time was 7.4 months, while that of those who didn’t receive it was 1.2 months. Nevertheless, surgical approaches, radiotherapy, age at presentation, gender, tumor location, and metastatic status were not associated with independent prognosis.

**Conclusion:**

ETANTR might not present as the typical morphologies during tumor progression, so analyses of *C19MC* amplification and Lin28A antibody are indispensable for diagnosing ETMRs accurately. Children aged > 4 years tend to have a higher rate of metastasis in ETMRs. Chemotherapy is the only prognostic factor for ETMRs patients with a favorable prognosis. The biological nature and clinical patterns for recurrent diseases need to be further demonstrated to predict prognosis and guide treatment.

## Introduction

Embryonal tumors with multilayered rosettes are a novel entity that is extremely rare and predominantly affects children aged ≤ 2 years old (1). In the 2007 WHO classification of the central nervous system (CNS), these tumors were not well classified and were still under the broad umbrella of the CNS-primitive neuroectodermal tumors (PNET) (2). Large-scale studies subsequently showed that ETANTRs, ependymoblastoma, and medulloepithelioma (ME) shared alterations in the *C19MC* locus at the Chr 19q13.42 (3–6). Therefore, these three histologic subtypes were merged into ETMRs in the 2016 WHO classification of tumors of the CNS (7), and the 2021 WHO also follows this classification standard (1).

Histologically, these subgroups share multilayered (ependymoblastoma) rosettes and small embryonal tumors. ETMRs could develop through the CNS but mainly involve the cerebral hemisphere (8, 9). They correspond to WHO grade 4 and are associated with an aggressive clinical course. However, standard and optimal treatment has not been established, and the effects of current therapies remain unclear in ETMRs (8, 10). Moreover, the histopathological features of ETMRs have not yet been demonstrated during tumor progression, including recurrence and metastasis. Our study aimed to review and collect the cases diagnosed with ETMRs in our hospital to identify the clinical and pathologic characteristics, pathogenesis, and diagnostic factors of ETMRs.

## Materials and methods

### Study cohort

Seventeen patients with ETMRs were diagnosed at our hospital from January 2018 to January 2022, including ETANTRs and medulloepithelioma. All diagnoses were made according to the 2021 WHO CNS tumor classification criteria and reviewed by two expertized neuropathologists. Surgery approaches were divided into biopsy, partial resection, and complete resection. Two patients were lost to follow-up; therefore, fifteen were included in our statistical analysis. Besides, we collected 31 patients with atypical teratoid/rhabdoid tumors (AT/RTs) and 50 patients with medulloblastomas (MB) as control groups.

### Immunohistochemistry and fluorescence *in situ* hybridization

Immunohistochemistry (IHC) was performed on 10% neutral buffered formalin-fixed, paraffin-embedded (FFPE) 4-um-thick tissue sections using the two-step Envision standard. The primary antibodies included: Lin28A (Cell Signaling Technology, USA), INI1 (BAF47/INI-1; Santa Cruz, USA), glial fibrillary acidic protein (GFAP; Dako, Denmark), oligodendrocyte transcription factor 2 (Olig2; Dako), synaptophysin (Syn; Novocastra, UK), neuronal nuclei (NeuN; Dako), and Ki-67 (Dako). Dual-color fluorescence *in situ* hybridization (FISH) using an intermittent microwave irradiation method was performed on 4-um-thick FFPE tissue sections (11). The *C19MC*/19q13.42 probe and a controlled 19p13.11 probe were prepared from bacterial artificial chromosome (BAC) clones as previously described (6). Lin28 IHC and INI1 IHC were performed on all ETMRs and controls, while other stains and *C19MC* FISH were performed on ETMRs.

### Statistical analysis

Log-rank analysis using the Kaplan Meier and Chi-square analysis was applied to compare survival time, including overall survival (OS) and event-free survival (EFS). Cox regression analysis was performed to identify independent predictors. The level of significance was *p* = 0.05. The statistical analyses were performed using SPSS 26.0 (IBM, USA) and Prism 9.1.1 (Graphpad Software, USA).

## Results

### Clinical characteristics and neuroimaging features

The clinical data of all 17 patients are listed in **Table 1**. The age at diagnosis ranged from 16 months to 55 months (median age, 29 months); 76% (13/17) of the patients were ≤ 4 years old, and the male: female ratio was 1.83:1. Progressive-sided weakness (6/17) and vomiting (6/17) were the main symptoms, while strabismus and convulsion occurred in 3 and 2 patients, respectively.

**Table 1:** Clinical details of 17 patients of ETMRs.

Number	Age (y)	Gender	Therapy	Chemotherapy	Radiotherapy	EFS (m)	OS (m)	outcome
1	2.7	F	Surg (Biopsy)	/	/	0.3	0.3	DOD
2	2.6	M	Surg (Biopsy) + RT + ChT	VCR + Carbo+ VP	32Gy CSI/22Gy focal boost	43	43	AWD
3^*^	2.0	M	Surg (PR) + RT + ChT	CTX + VCR	36Gy CSI/54Gy focal boost	5.3	6.3	DOD
4^*c^	3.4	F	Surg (CR) + RT + ChT	CTX + DDP + VCR	54Gy focus	6.5	9.5	DOD
5^*^	4.2	M	Surg (PR)	/	/	0.9	1.2	DOD
6^*^	4.3	F	Surg (CR) + RT + ChT	CTX + DDP + VCR	54Gy focus	4.8	16	DOD
7^*^	4.6	M	Surg (PR)	/	/	0.8	0.9	DOD
8^c^	1.9	M	Surg (CR)	/	/	1.3	1.4	DOD
9	2.0	F	Surg (PR)	/	/	0.8	0.8	DOD
10	1.3	M	Surg (CR) + ChT	CDDP + VCR + VP + CTX + MTX	/	5.8	5.8	DOD
11^*^	4.1	M	Surg (CR) + RT	/	54Gy focus	7.0	8.9	DOD
12	1.9	M	Surg (PR) + ChT	VCR + Carbo + VP	/	1.4	1.4	AWD
13	2.1	M	Surg (CR) + ChT	VCR + Carbo + VP	/	0.6	0.6	NED
14	2.2	F	Surg (Biopsy)	/	/	1.2	1.2	DOD
15	2.5	M	Surg (PR) + ChT	VCR + Carbo+ VP	/	8.5	8.5	DOD
6^ac^	1.4	F	Surg (PR)	lost	lost	lost	lost	lost
17^b^	3.6	M	Surg (Biopsy)	lost	lost	lost	lost	lost

/: patients did not receive the treatment; n^*^, patients occurred with intracranial or spinal metastasis; n^C^, patients occurred with relapse; 16^a^ and 17^b^ were patients who were lost to follow-up.

DOD, die of disease; AWD, alive with disease; NED, no evidence of disease; y, years; m, months; Surg, surgery; PR, partial resection; CR, complete resection; ChT, chemotherapy; RT, radiotherapy; OS, overall survival; EFS, event-free survival.

**Figure 1:**
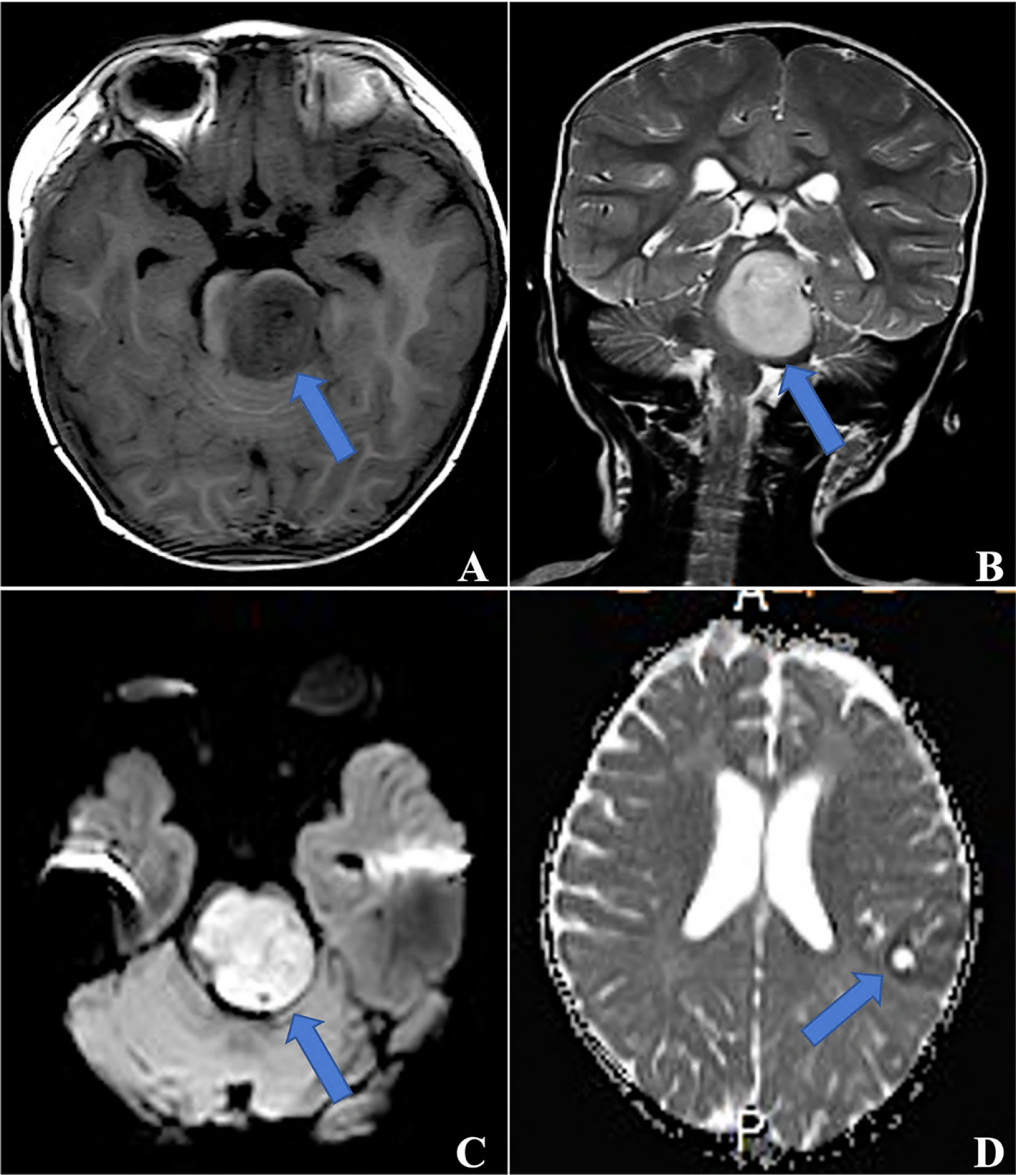
MRI for ETMRs, **(A)** TIWI showed a hypointense mass in the brainstem, which was hyperintense on both T2WI **(B)** and DWI **(C)** (blue arrows, case 5); **(D)** a 3×2×1 mass in the left parietal and occipital lobes with ringlike contrast enhancement (blue arrow, case 6).

The initial site was supratentorial in 10 patients and infratentorial in 7 patients, with a ratio of 1.4:1. Initial magnetic resonance imaging (MRI) information was available for 15 cases, and the other two were unavailable as MRI was performed at a local hospital. In 15 cases, tumors were isointense or hypointense on T1-weighted imaging (T1WI) and isointense or hyperintense on T2 WI. In contrast, tumors exhibited slight or partial contrast enhancement and hyperintensity on diffusion-weighted imaging (DWI) **(** **Figure 1** **)**. Three of these tumors contained single or multiple partially cystic appearances. Calcification was not found in our cohort.

### Histopathological features

**Figure 2:**
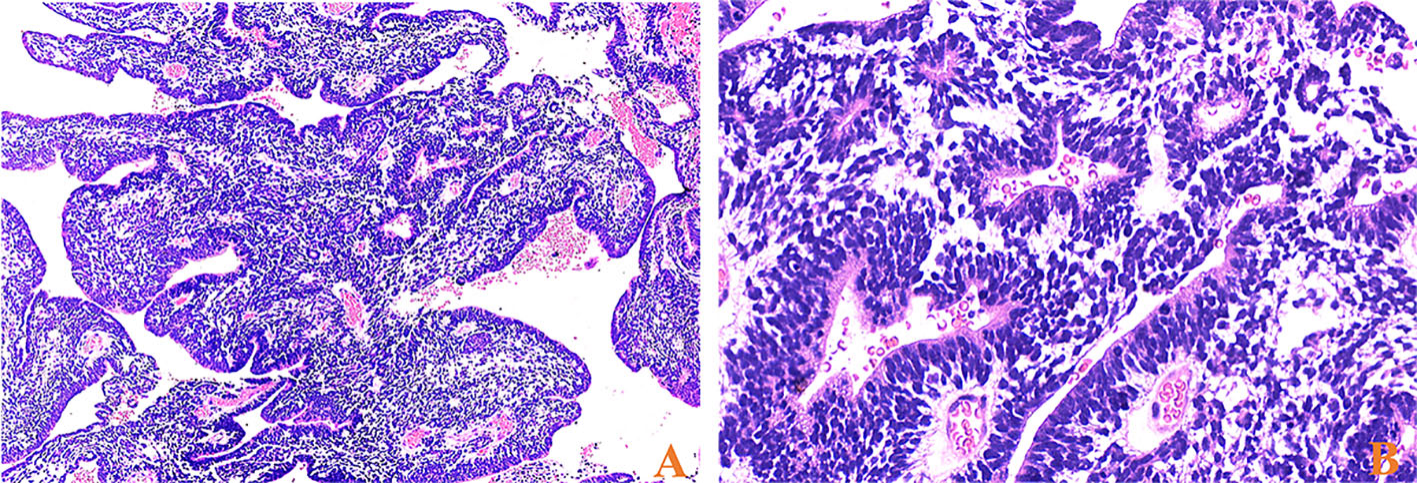
HE stainings of medulloepithelium for case 9, **(A)** papillary-like and tubular structures resembling the primitive neural tube (×40) and **(B)** on the luminal surface of these tubules, cilia, and blepharoplasts were absent (×400).

**Figure 3:**
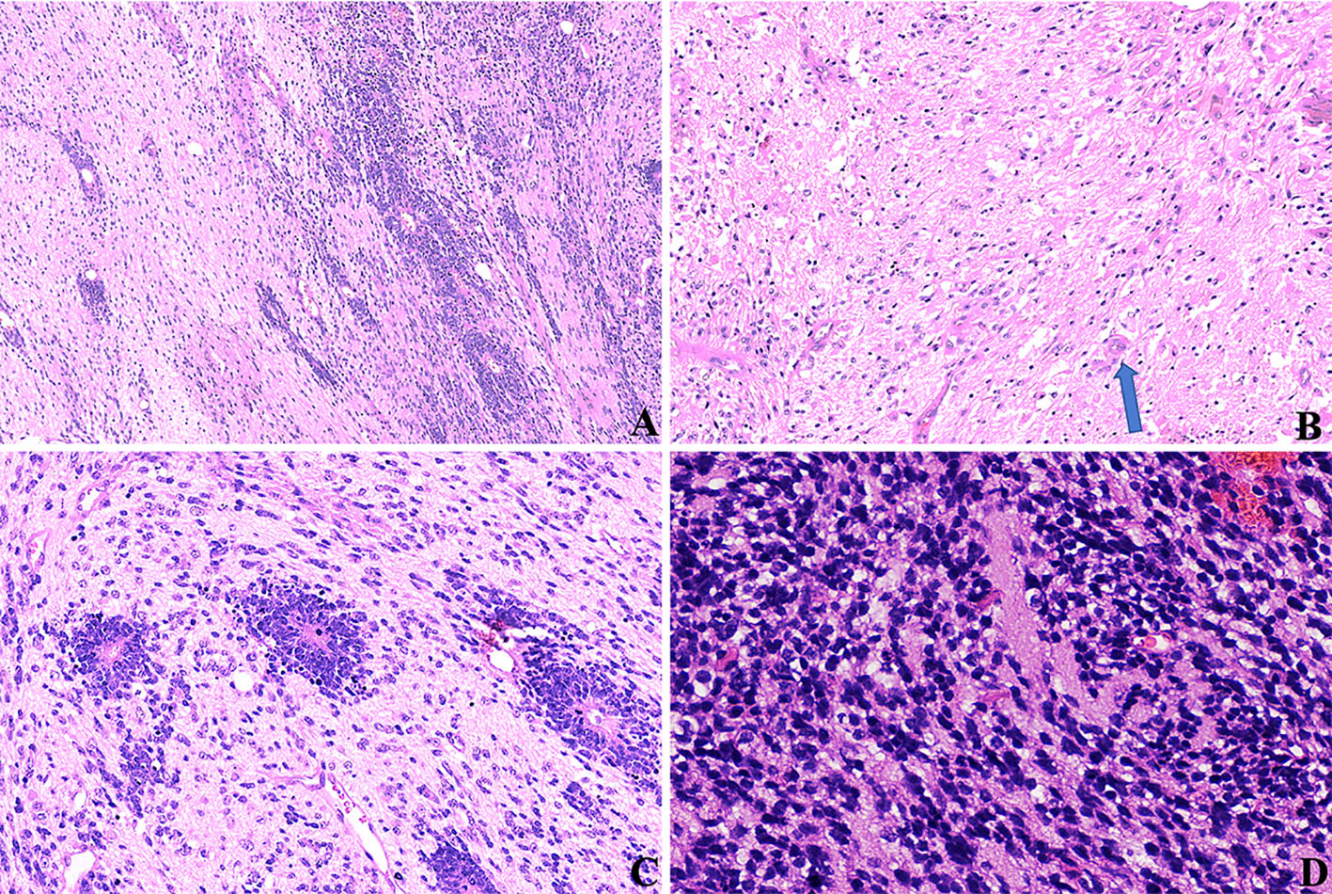
HE stainings of ETMRs for case 1, **(A)** tumor cells were in a biphasic architecture featuring dense clusters of mall embryonal cells/multilayered rosettes and paucicellular neuropil-like areas (×100); **(B)** Sparse neoplastic neurocytic and ganglion cells could be observed in the neuropil-like matrix (blue arrow, ×200); **(C)** Multilayered rosettes comprised embryonal cells in a pseudostratified neuroepithelium with a central round or slit-like lumen; The cells facing the lumen have a defined apical surface with a prominent internal limiting membrane; The nuclei of the rosette-forming cells tended to be located away from the lumen towards the outer cell border (×200); **(D)** small embryonal cells were with round or polygonal nuclei and scant cytoplasm (×400).

**Figure 4:**
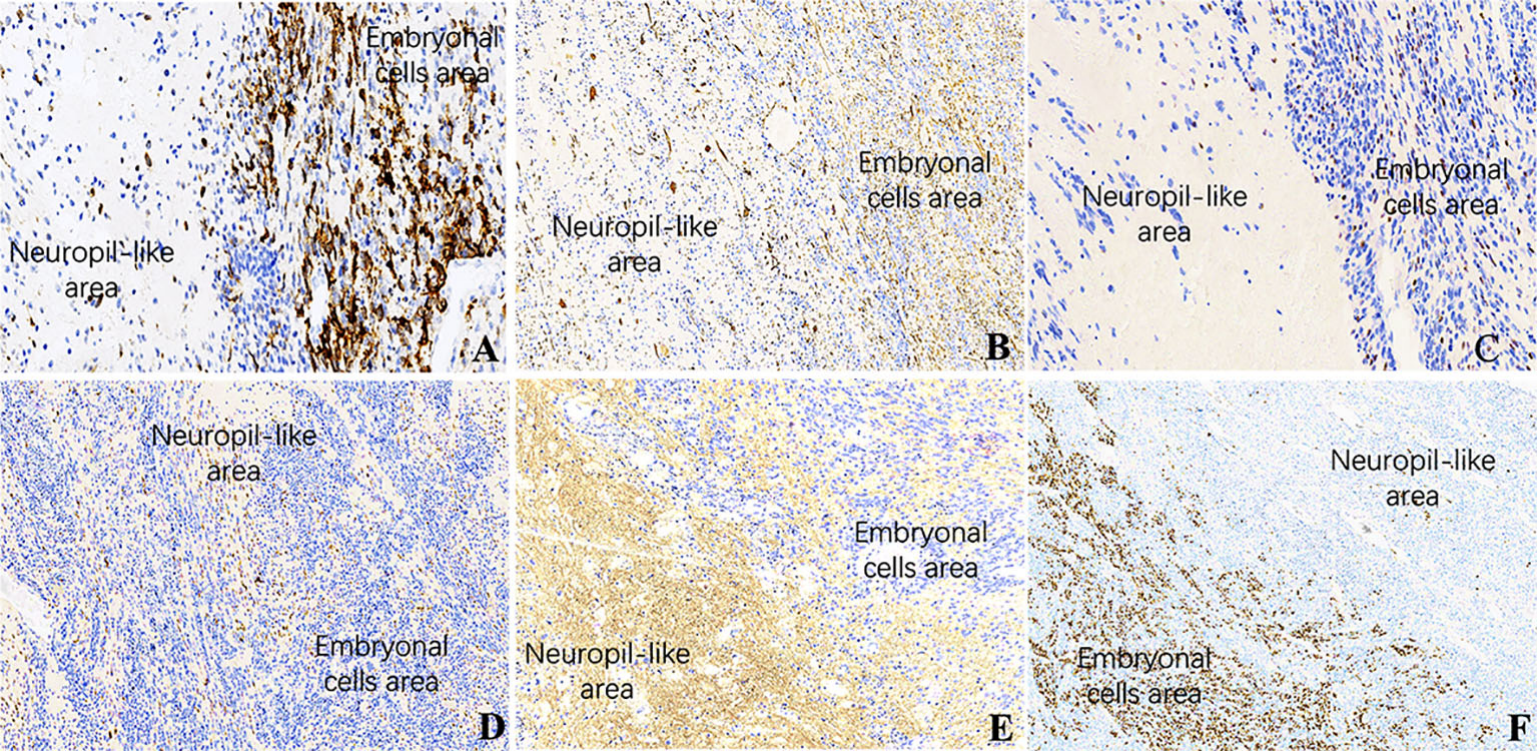
IHC of ETMRs for case 1, the expressions of **(A)** LIN28A, **(B)** GFAP, **(C)** OLIG2, and **(F)** Ki67 were stronger and more diffuse in the embryonal cell areas than in the neuropil-like matrix (×200); Neuronal makers, including NeuN **(D)** and Syn **(E)**, expressed in the neuron differentiated cells but were negative in embryonal cells (×200).

**Figure 5:**
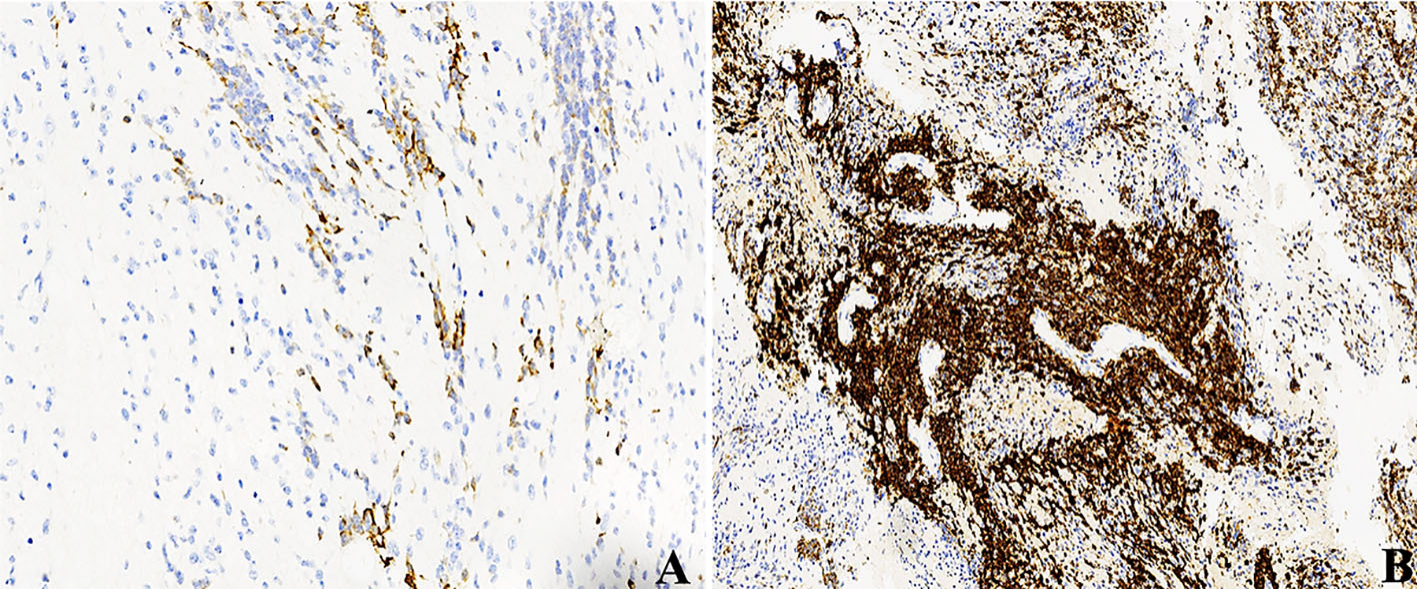
LIN28A IHC in primary and relapsed ETMRs for case 8, **(A)** LIN28A cytoplasmic immunopositivity was weaker and focal in primary tumors compared with **(B)** in recurrent counterparts (×200).

**Figure 6:**
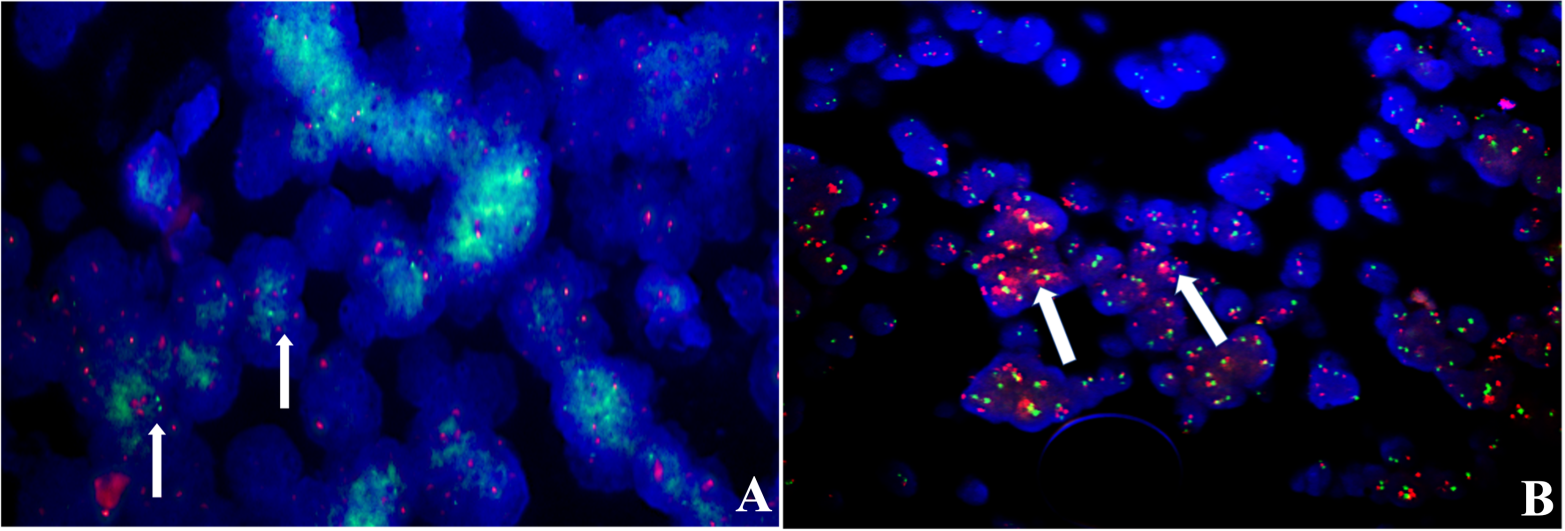
FISH analysis, **(A)** *N-MYC* for relapsed case 8 showed increased copy numbers, green signals (white arrows); **(B)** case 12 showed amplification in the GSP *C19MC* probe, red signals (white arrows).

Among the 17 patients, tumor sizes varied from 0.5 to 10cm, with a median size of 2.5cm. Sixteen patients had ETANTRs, 1 had medulloepithelioma (case 9), and no ependymoblastoma. The pathological features of the 17 cases are listed in **Table 2**. Morphologically, medulloepithelium was characterized by papillary, tubular, and trabecular arrangements **(** **Figure 2** **)**, as well as sheets of poorly differentiated cells. ETANTRs showed biphasic architecture featuring dense clusters of small embryonal cells (or multilayered rosettes) and paucicellular neuropil-like areas **(** **Figure 3** **)**. In ETANTRs, the neuropil-like areas were stained strongly and diffusely for the neuronal markers such as NeuN and Syn. In contrast, only a few positive cells were found within the multilayered rosettes and small cell areas **(** **Figures 4D, E** **)**. The glial markers GFAP and Olig2 focally expressed in some embryonal cells but were negative in the neuron differentiated cells **(** **Figures 4B, C** **)**. The Ki67 index ranged from 40% to 90%, which was relatively high in embryonal cells and lowered in the neuropil-like areas **(** **Figure 4F** **)**. All tumors demonstrated diffused nuclear expression of INI1 throughout all components. Lin28A expression was found in all cases (17/17), and its immunoreactivity was more predominant and intense in multilayered rosettes and poorly differentiated small cell areas compared with neuropil-like regions **(** **Figure 4A** **)**. In our three relapsed cases, the expression of LIN28A was much stronger and more intense in the recurrent lesions compared with primary tumors **(** **Figure 5** **)**. Conversely, tumor cells of AT/RTs were partially or focally positive for Lin28A in13 cases (13/31, 42%), but all cases showed a nuclear loss of INI-1. In 50 cases of medulloblastoma, all tumor cells were negative for Lin28A and positive for INI-1. Besides, the increased copy number of *MYC* (*N*) was found in case 8 of both primary and recurrent lesions (**Figure 6A**), whereas no *NRAS* and *CDK6* aberrations were detected (**Table 3**). The amplification of the locus 19q13.42, analyzed in 17 ETMRs, was presented in 16 patients **(** **Figure 6B** **)**. For the patient without the amplification of *C19MC* (case 9), we performed the FISH analysis of *DICER1*, and mutations were not detected.

**Table 2:** Histopathologic features of 17 patients of ETMRs.

Number	Morphology	Mitoses/10 HPFs	Ki67 index	LIN28A IHC	*C19MC* amplification	GFAP	NeuN	Specimen obtained method
1	ETANTR	8	40%	+	yes	–	+	at diagnose
2	ETANTR	15	60%	+	yes	+	–	at diagnose
3^*^	ETANTR	35	80%	+	yes	+	+	at diagnose
4^*c^	ETANTR	28 (primaries) 30 (relapse)	60% (primaries) 80% (relapse)	+	yes	+	+	at diagnose
5^*^	ETANTR	38	90%	+	yes	+	+	at diagnose
6^*^	ETANTR	30	80%	+	yes	+	–	at diagnose
7^*^	ETANTR	39	70%	+	yes	+	+	at diagnose
8^c^	ETANTR	35 (primaries) 37 (relapse)	70% (primaries) 75% (relapse)	+	yes	+	+	at diagnose
9	ME	28	80%	+	no	+	+	at diagnose
10	ETANTR	39	40%	+	yes	+	–	at diagnose
11^*^	ETANTR	32	80%	+	yes	+	–	at diagnose
12	ETANTR	26	70%	+	yes	+	+	at diagnose
13	ETANTR	45	60%	+	yes	+	+	at diagnose
14	ETANTR	42	70%	+	yes	+	–	at diagnose
15	ETANTR	36	80%	+	yes	+	–	at diagnose
16^ac^	ETANTR	15 (primaries) 35 (relapse)	40% (primaries) 50% (relapse)	+	yes	+	+	relapse
17^b^	ETANTR	30	80%	+	yes	+	+	at diagnose

/: patients did not receive the treatment; n^*^: patients occurred with intracranial or spinal metastasis; n^C^: patients occurred with relapse; 16^a^ and 17^b^ were patients who were lost to follow-up.

ETANTR, embryonal tumors with abundant neuropil and true rosettes; ME, medulloepithelioma.

**Table 3:** Pathological and molecular features of case 8.

markers / lesions	KI67	P53 IHC	*TP53* gene	*N-MYC*	*NRAS*	*CDK6*
primary	70%+	–	wild-type	increased copy numbers	no mutation	no amplification
relapsed	75%+	–	wild-type	increased copy numbers	no mutation	no amplification

**Figure 7:**
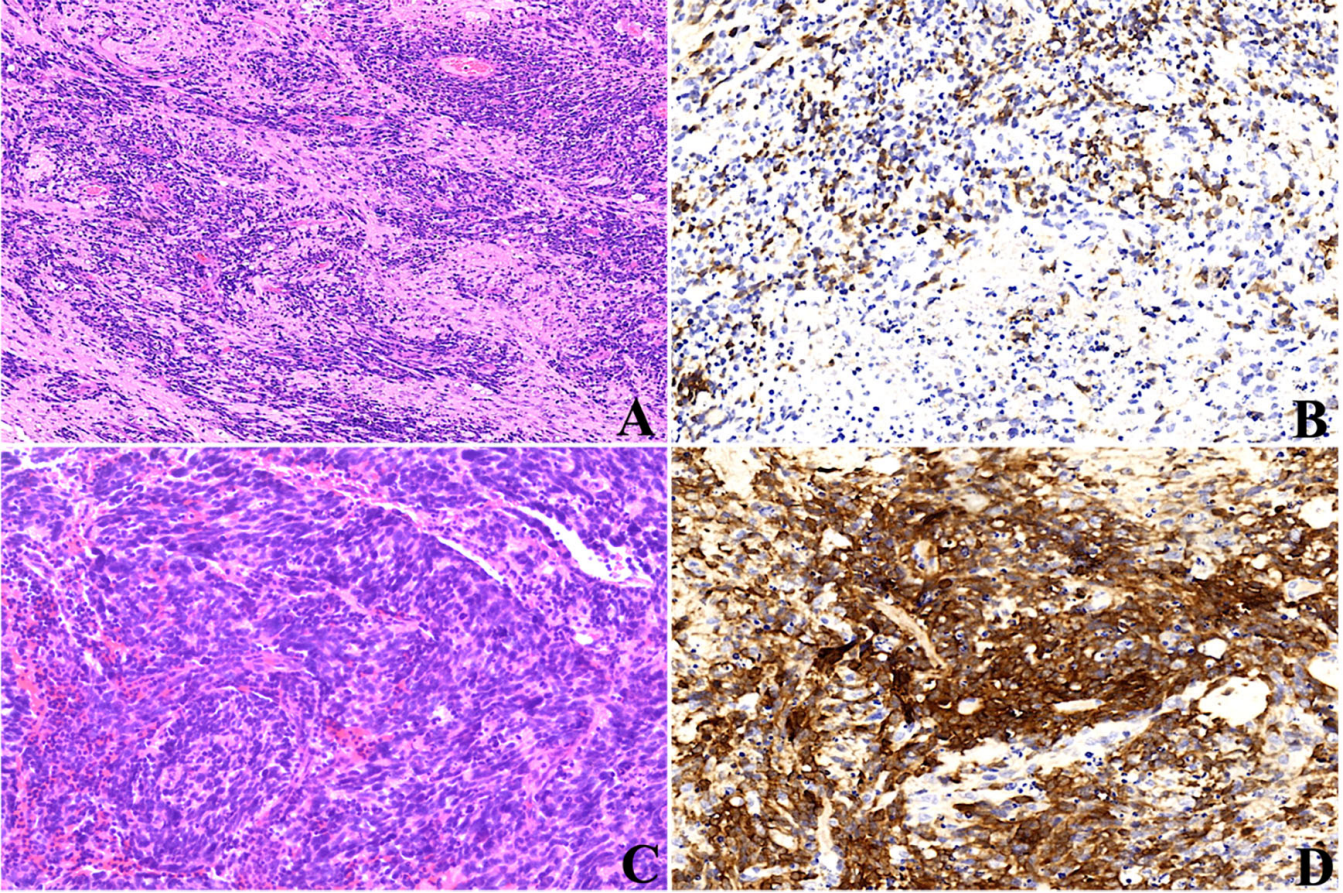
Histopathological progression of case 4, the tumor presented as the biphasic pattern of ETANTRs at the initial surgery **(A)**, and Lin28A expression was positive **(B)**; recurrent tumor after surgery showed anaplasia in large cells with cellular pleomorphism and prominent nucleoli **(C)**; the expression of Lin28A was stronger and more intense **(D)** than that of the primary lesion.

One case (1/8) occurred with the histological evolution towards anaplasia during tumor progression (case 4). At first surgery, histopathology revealed a biphasic architecture featuring small embryonal cells with round or polygonal nuclei and scant cytoplasm and neuropil-like areas with sparse neoplastic neurocytic and ganglion cells **(** **Figure 7A** **)**, confirming the diagnosis of ETMR. IHC showed the diffuse expression of Lin28A **(** **Figure 7B** **)** and focal expression of SYN and NeuN in tumor cells. FISH analysis revealed the amplification of *C19MC.* After 6.5 months, the tumor presented with recurrence demonstrated by MRI and undertook the complete resection. Histopathology showed tumor cells were three times larger than their neighboring cells with cellular pleomorphism and prominent nucleoli, indicating the anaplastic progression, but neither neuropils nor ependymoblastic rosettes were observed **(** **Figure 7C** **)**. Amplification at 19q13.42 and immunopositivity of LIN28A **(** **Figure 7D** **)** still existed in the tumor cells, which demonstrated the recurrence of ETMRs.

### Treatment and outcome

All 17 patients received surgery, with five undergoing complete resections, four biopsies, and eight partial resections. Two patients were lost to follow-up. Therefore, therapy and follow-up data were only available for 15 patients **(** **Table 1** **)**.

Five patients occurred with metastatic disease, two with recurrence, and one presented with both metastasis and recurrence. Among the six patients with metastatic disease, one developed spinal metastasis from the fourth ventricle, and two occurred with intracranial metastasis at initial presentation. All the metastatic lesions were visible by MRI, while no metastatic cells were found in the cerebrospinal fluid. The 2021 WHO indicates that ETMRs occur in children ≤ 4 years of age, and analyses performed with stratification for periods ≤ and > 4 years showed that two patients had intracranial metastasis in the younger group (18%, 2/11) and four in the elder group (100%, 4/4). The incidence of metastasis was significantly different between the two groups (*p* = 0.025, χ2 = 9.76; **Table 4**), demonstrating that children aged > 4 years were prone to occur with metastasis.

**Table 4:** Correlations between metastatic status and age in ETMRs.

age (years) / Metastatic status	aged ≤ 3 (n = 11)	aged > 3 (n = 6)	*X^2^* = 9.97 *P* = 0.002
metastasis (n = 6 )	1	5
without metastasis (n = 6 )	10	1
Total (n = 17)	11	6

**Figure 8:**
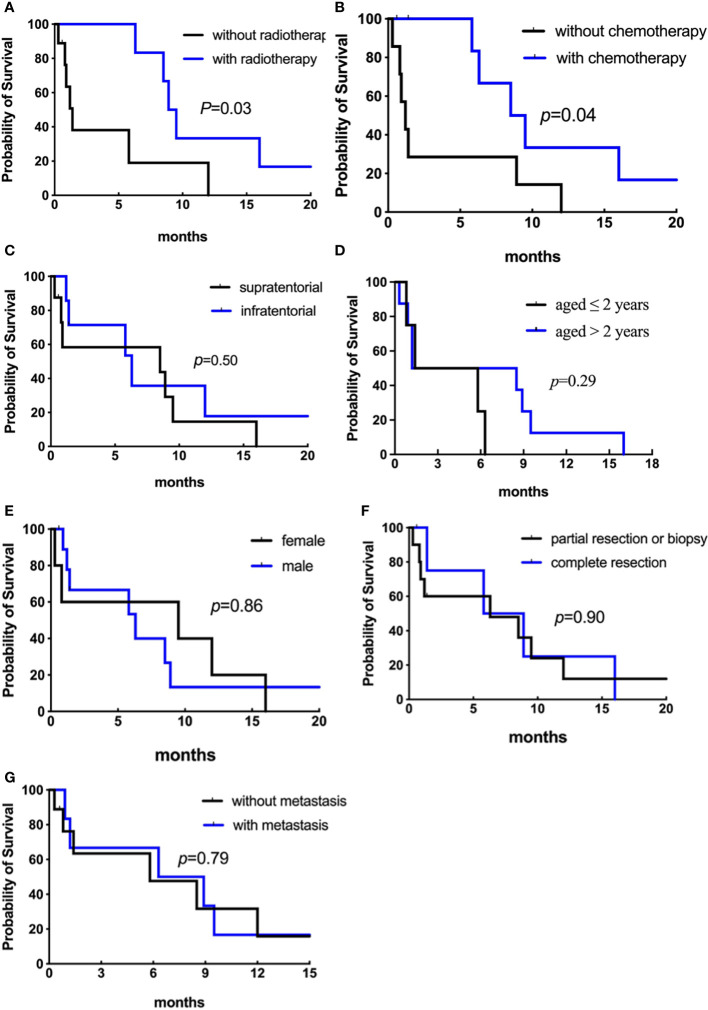
Analyses of overall survival (Kaplan–Meier method) according to **(A)** radiotherapy (*p* = 0.03); **(B)** chemotherapy (*p* = 0.04); **(C)** primary tumor location (*p* = 0.50); **(D)** age at diagnosis (*p* = 0.29). Figure 8.2 Analyses of overall survival (Kaplan–Meier method) according to **(E)** gender (*p* = 0.86); **(F)** surgical resection (*p* = 0.90); **(G)** metastatic status (*p* = 0.79).

**Figure 9:**
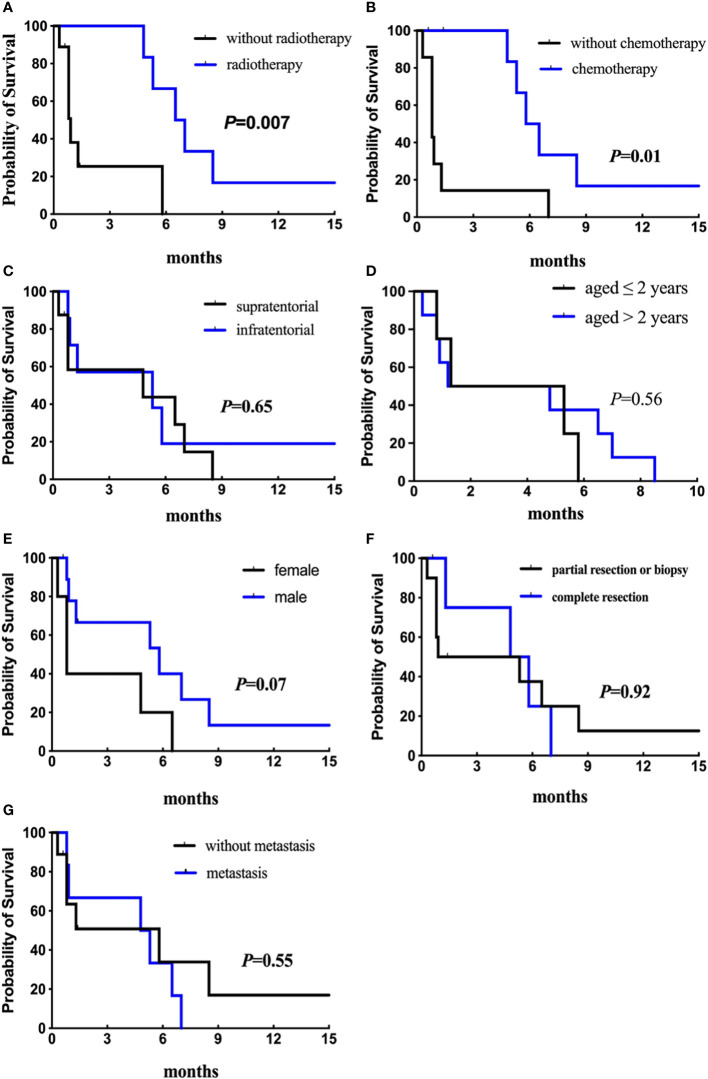
Analyses of event-free survival (Kaplan–Meier method) according to **(A)** radiotherapy (*p* = 0.007); **(B)** chemotherapy (*p* = 0.01); **(C)** primary tumor location (*p* = 0.65); **(D)** age at diagnosis (*p* = 0.56). Figure 9.2 Analyses of event-free survival (Kaplan–Meier method) according to **(E)** gender (*p* = 0.07); **(F)** surgical resection (*p* = 0.92); **(G)** metastatic status (*p* = 0.55).

Among the 15 patients, only three are alive, and the other 12 died of tumor progression. Six patients were without adjuvant therapy after initial surgery and died within a short time (0.3-1.3 months). Nine patients were treated with adjuvant treatment following surgery (1 biopsy, 3 partial, and 5 total resections); one patient received radiotherapy alone, four received chemotherapy alone, and four received radiotherapy plus chemotherapy. The median durations of OS and EFS were both 1.4 months. Analyses of OS and EFS showed patients who were treated with radiation treatment exhibited a higher survival rate than those who were not (*p* = 0.03 for OS, **Figure 8A**; *p* = 0.007 for EFS, **Figure 9A**). However, patients who underwent complete resection didn’t differ significantly from their partial resection or biopsy counterparts (*p* = 0.90 for OS, **Figure 8F**; *p* = 0.92 for EFS, **Figure 9F**). Additionally, tumor location, age, gender, and metastasis were not significantly associated with OS or EFS (respectively for OS, *p* = 0.50, *p* = 0.29, *p* = 0.86, *p* = 0.79, **Figures 8C, D, E, G**; respectively for EFS, *p* = 0.65, *p* = 0.56, *p* = 0.07, *p* = 0.55, **Figures 9C, D, E, G**).

The types of chemotherapeutic agents in our study were different between patients with and without complete resection, including vincristine, carboplatin, prednisone, cyclophosphamide, and cisplatin (see **Table 1**). Eight patients received chemotherapy, and one experienced long-term survival of greater than 36 months. Among them, the median OS and EFS were 7.4 and 5.6 months, respectively, while that of the patients without chemotherapy were 1.2 and 0.8 months, respectively (*p* = 0.04 for OS, **Figure 8B**; *p* = 0.01 for EFS, **Figure 9B**). We then removed age and other potential treatment biases on survival, and the multivariate analysis revealed that chemotherapy with a relative risk (RR) of 0.172 CI 95% (0.032-0.910) *p* = 0.038 was the only prognostic factor associated with better overall survival.

## Discussion

ETMRs are extremely rare, and their true incidence is difficult to calculate; about 100 cases have been reported in the English medical literature since its first description in 2000 by Eberhart et al. (12). However, some patients may have been misdiagnosed or missed before its defining genetic alteration was established. The tumor mainly affects infants aged < 4 years old, and 76% of patients were under the age of 4 years in our analysis, but patients aged > 18 years have also been reported (10). Our data indicated that the male-to-female ratio was 1.8:1, which is consistent with the male predominance in CNS embryonal tumors. Supratentorial tumors are more often than infratentorial tumors (6, 8, 13, 14), as observed in our cohort, while rare cases arising in the spine have been reported (8, 15). On neuroimaging, these tumors were demarcated and large. MRI demonstrated that tumors were usually hypointense on T1WI and hyperintense on T2WI and DWI, with minimal or partial contrast enhancement (16, 17 ,13). Tumors may contain cysts or calcification (18). Of note, the imaging features of ETMRs are similar to that of AT/RTs and MB. Nevertheless, accurate diagnosis is crucial due to their distinct treatment protocols and biology, leading to a dismal prognosis of ETMRs that differs significantly from AT/RTs and MB. The average OS of ETMRs after intensive therapies is approximately 12 months (6, 8, 13), compared with a 37% of 4-year OS rate in AT/RTs (19) and the 5-year survival rate of MB is 85% (20).

Histologically, poorly differentiated small cells and multilayered (ependymoblastic) rosettes are the characteristic and standard features of ETMRs (12, 13, 21). Some tumor cells in ETMRs exhibit osteoid, myeloid, epithelial, mesenchymal, or muscular differentiation or contain cytoplasmic melanin pigment (21, 22). The morphological progression between the primary and recurrent lesions in patient 4 indicates the diagnostic histology of ETMRs is not always present and might result in misdiagnosis. Tumor progression towards anaplasia may be caused by genetic aberrations like *TP53* (13, 23, 24), and it is interesting to consider whether anaplasia is of prognostic significance (as it is for medulloblastoma). However, more case numbers will need to be collected to explore it. Besides, glial and neuronal differentiation/maturation in ETMRs after combination therapies have been reported, and the significance of treatment and prognosis remains to be further demonstrated (25). Therefore, the analyses of *C19MC* amplification and Lin28A immunoreactivity are indispensable and should be performed in all CNS embryonal tumors in children to diagnose of ETMRs accurately. Though *C19MC* alterations are specific for ETMRs, LIN28A immunopositivity is not. Our result revealed that all cases showed positive expression of LIN28A while 16 cases had altered *C19MC*.

Immunohistochemical staining for Lin28A is more intense and prominent in the small cells and multilayered rosettes compared with neuron-differentiated cells in ETMRs irrespective of histological subgroups (13, 26). In the present study, the staining for Lin28A was more diffuse and prominent in the relapsed lesions than in primaries, which is supported by Andrey (27) as the recurrent ETMRs may be composed of hypercellular but lacked neuropil areas. Lin28A immunopositivity is not unique to ETMRs (10, 27); our study found it was also expressed focally in AT/RTs but was negative in MB. According to a large study, the strong expression of Lin28A in ETMRs statistically relates to poorer outcomes than those of negative or focal expression in other CNS embryonal tumors (27). Collectively, Lin28A IHC is of both diagnostic and prognostic significance in ETMRs (28, 29). The limitation of our study is that we didn’t compare the prognosis of ETMRs with the control groups.

The origin cells and biology of ETMRs remain poorly elucidated. Recently, the *C19MC* amplification at the Chr 19q 13.42 and the LIN28A/Let-7 signaling pathways are considered crucial hallmarks for tumorigenesis (5, 26, 27). 90% of ETMRs are characterized by amplification of the polycistronic microRNA (miRNA) cluster *C19MC* on chromosome 19q13.42, one of the largest miRNA clusters that contain 60 kb and 46 miRNA genes (30, 31). Compared with supratentorial ETMRs, infratentorial tumors typically have a higher frequency of *C19MC* amplification. Approximately half of the *C19MC*-negative ETMRs, representing 5% of all ETMRs, harbor biallelic *DICER1* mutations and are mutually exclusive to *C19MC* amplification. Moreover, ETMRs characteristically express the RNA binding protein LIN28A, which could downregulate the let-7 family of miRNAs and is further associated with activating mTOR, WNT, and *MYCN* signaling pathways involving oncogenesis (27, 32). Of note, the LIN28A/let-7 pathway is the common downstream of alterations of *C19MC* and *DICER1*, which *C19MC* may affect LIN28A indirectly by downregulating Tristetraprolin (TTP). At the same time, WNT and *MYCN* signaling are downstream of the RNase III domain in *DICER1* mutations, so ETMRs are molecularly similar whether they have *C19MC* amplification (33–35). Our case 9, which had a tumor in the frontal lobe, harbored neither *C19MC* amplification nor *DICER1* mutation and should be classified as ETMR, not elsewhere classified (NEC), according to the 2021 WHO CNS classification (7). One explanation is that other genetic abnormalities, such as amplification of the miR17HG on chromosome 13, might drive the tumorgenesis of ETMRs, NEC (26). Moreover, our data showed the aberrant expression of *N-MYC* in case 8, which might play a role in tumor relapse (27), and the molecular genotyping didn’t change at recurrence. Therefore, more cases and research are needed to explore the biological nature and clinical patterns of the recurrent diseases to guide the therapy better.

To date, no standard therapeutic approaches have been established for ETMRs. The treatment now includes surgery, radiotherapy, and chemotherapy, but the prognosis of ETMRs remains poorer. In our analysis, the extent of surgical resection was not associated with overall survival. This result was different from the finding reported by Kirti et al. (10), in which total resection was a positive indicator of survival in ETMRs. The limitation of our study lies in that the extent of resection was not quantitated exactly. However, it indicated that there is no obligation for surgeons to conduct the total resection of the tumor, and biopsy is adequate for pathologic diagnosis and avoiding neurocognitive impairment. Besides, we found radiotherapy was not the prognostic factor in a multivariate analysis, similar to Alexiou‘s research (36). However, Horwitz et al. (8) found radiotherapy was associated with a better outcome using both univariate and multivariate analyses. The bias in the literature may be introduced by the fact that radiotherapy was treated after chemotherapy and surgery, which could screen patients. The radiation volumes have not been identified in ETMR; as the younger age in the ETMRs population (median age: 2 years), radiotherapy should be administrated with caution because of the late effects. Our findings revealed that radiotherapy might not be recommended for ETMRs and could decrease neurocognitive impairment. Due to the limitation of tumor samples, the prognosis was not compared among the three subtypes of ETMRs.

The effectiveness and prognostic role of chemotherapy have not been reliably identified hitherto. Due to the lack of established protocols, the type of chemotherapeutic agents varies among patients. Our data found that patients who received chemotherapy tend to have a statistically better prognosis than those who weren’t treated with chemotherapy, similar to some findings (8, 37). The favorable prognosis of chemotherapy is possible because it accelerates the neuron maturation of tumor cells (37). However, long-time OS of chemotherapy has not been identified in both our cohort and literature as a result of the small sample size and short duration. Therefore, more cases and long-time follow-ups are needed to explore the survival data of chemotherapy. Moreover, our results revealed that patients aged > 4 years had a higher likelihood of metastasis, as shown in other CNS embryonal tumors (38). One explanation is that tumor progression (i.e., tumor spread through the neuraxis) in an older child simply because cancer has had (presumably) more time to grow and disseminate.

Due to the limited effects of the current therapeutic strategy for ETMRs, novel treatment options are needed. Tara et al. (5) found that the ETMRs cell line, BT183, is more sensitive to inhibitors of the IGF/PI3K/mTO*R* pathway. Another study proposed that *SHH* inhibitors could benefit ETMRs by activating the WNT pathway and upregulating the SHH pathway (39). Large-scale research and rigorous preclinical testing are required to support these drug targets to guide the molecularly targeted medicine further. Though LIN28/let-7 pathway is a possible promising target, the mechanisms among *C19MC* amplification, *DICER1* mutations, and LIN28 expression remain unclear. A more in-depth understanding of tumor biology is needed as well in ETMRs.In conclusion, our study covers the impressive number of patients diagnosed with ETMRs in a short time frame. Our data revealed that children over four years old have a higher rate of metastasis. The morphology of ETMRs varies, and characteristic features are not always presented along with tumor progression, which reminds us of the combination of the *C19MC* FISH and LIN28A IHC when making the diagnosis. Patients treated with chemotherapy tend to have a better prognosis. Since *C19MC* aberration has been detected for more than two decades, the understanding of ETMRs biology has improved as well as better diagnostic tools, more driving aberrations, and possible downstream targets. However, the survival of ETMRs patients has only minimally improved due to the lack of effective therapies; therefore, more case numbers and further studies are needed to demonstrate the involved signaling pathways and develop targeted molecular therapies.

## Data availability statement

The raw data supporting the conclusions of this article will be made available by the authors, without undue reservation.

## Ethics statement

The studies involving human participants were reviewed and approved by the Research Ethics Committee of the Shanghai Jiao Tong University School of Medicine. Written informed consent to participate in this study was provided by the patient/participants legal guardian/next of kin.

## Author contributions

KX contributed to the investigation and was a significant contributor to writing the manuscript; ZS designed the project and collected data; LW performed the data analysis and designed experiments; WG revised the manuscript; KX and ZS contributed equally to the work and share first authorship. All authors read and approved the final manuscript.

## Conflict of interest

The authors declare that the research was conducted in the absence of any commercial or financial relationships that could be construed as a potential conflict of interest.

## Publisher’s note

All claims expressed in this article are solely those of the authors and do not necessarily represent those of their affiliated organizations, or those of the publisher, the editors and the reviewers. Any product that may be evaluated in this article, or claim that may be made by its manufacturer, is not guaranteed or endorsed by the publisher.
